# Histopathological profile of endometrium among peri and post-menopausal women with abnormal uterine bleeding and its correlation with endometrial thickness by transvaginal sonography: a retrospective study

**DOI:** 10.1186/s13000-025-01717-z

**Published:** 2025-11-10

**Authors:** Vichitra S, Ranjini Kudva

**Affiliations:** Manipal Academy of Higher Education, Manipal, India

**Keywords:** Abnormal uterine bleeding, Endometrial thickness, Histopathology, Transvaginal sonography, Perimenopause, Postmenopause

## Abstract

**Background:**

Abnormal uterine bleeding (AUB) is a prevalent clinical concern, particularly in women approaching or beyond menopause. With a myriad of possible etiologies ranging from benign hyperplasia to malignant transformations, accurate diagnosis becomes crucial. This study aims to examine the histopathological patterns of the endometrium in peri- and postmenopausal women presenting with abnormal uterine bleeding and correlate these findings with endometrial thickness (ET) measured by transvaginal sonography (TVS).

**Methods:**

A retrospective cohort of 307 women aged 40 and above presenting with abnormal uterine bleeding was evaluated over a year period. Clinical history and transvaginal sonography findings were meticulously recorded. Endometrial samples obtained through biopsy or curettage were studied. The correlation between endometrial thickness and histological diagnosis was statistically analyzed using Mann-Whitney U Test and Chi square test, with a focus on distinguishing functional, benign, pre-malignant, and malignant endometrial pathologies.

**Results:**

Endometrial polyp is the most frequent pattern in both perimenopausal and postmenopausal women. An ET > 11 mm in peri menopausal and postmenopausal women showed a strong association with hyperplasia and malignancy. This suggests that transvaginal sonography, as a non-invasive tool, can significantly guide diagnostic and management strategies when interpreted alongside clinical and histopathological parameters.

**Conclusion:**

Endometrial thickness serves as a valuable adjunct in the evaluation of abnormal uterine bleeding. However, the gold standard for a conclusive diagnosis is endometrial tissue biopsy. Integrating histopathology with imaging findings enhances diagnostic precision, allowing early identification of precancerous and cancerous lesions, especially in the postmenopausal cohort. Our study concludes that endometrial sample is recommended when ET > 11 mm in perimenopausal and ET > 5 mm in postmenopausal women, particularly when bleeding is persistent.

## Background

Abnormal uterine bleeding (AUB) represents one of the most common gynecological concerns, particularly affecting women transitioning through perimenopause and those who are postmenopausal. A typical menstrual cycle lasts about 4 ± 3 days at intervals of 28 ± 7 and 30-80 ml of blood is lost per cycle. Any deviations in frequency, volume, or duration constitute abnormal patterns that warrant further evaluation [1]. AUB accounts for approximately 33% of outpatient visits in gynecology and remains a major indication for endometrial assessment, especially in the post- menopausal group [1].

In perimenopausal women, hormonal fluctuations and anovulatory cycles frequently manifest as irregular bleeding patterns. This is attributable to diminished ovarian follicular function and fluctuating estradiol levels, resulting in endometrial instability [2]. Conversely, in postmenopausal women, AUB is considered pathognomonic until proven otherwise, often warranting exclusion of malignancy as the foremost priority [3]. Endometrial carcinoma remains a significant concern, with a global incidence of nearly 9 per 100,000 women, and is frequently preceded by a history of AUB [4].

The causes of AUB are diverse and span both structural and non-structural domains. To streamline diagnostic evaluation and communication, the International Federation of Gynecology and Obstetrics (FIGO) introduced the PALM-COEIN classification system, which categorizes etiologies based on structural (Polyp, Adenomyosis, Leiomyoma, Malignancy) and functional (Coagulopathy, Ovulatory dysfunction, Endometrial, Iatrogenic, Not otherwise classified) criteria [3, 5]. While histopathological examination of endometrial tissue remains the gold standard for diagnosis, it is inherently invasive and often not the initial approach.

To address this limitation, transvaginal ultrasonography (TVS) has emerged as a first-line, non-invasive imaging modality that can reliably measure endometrial thickness (ET) [6, 7]. Several guidelines, including those from the American College of Obstetricians and Gynecologists (ACOG), recommend TVS for initial assessment, particularly when ET measurements fall below 4 mm in postmenopausal women, given its high negative predictive value [8].

Despite its widespread adoption, there is a paucity of data defining standardized ET cut-off values in the perimenopausal population. Studies have variably suggested a threshold of 10–11 mm to prompt biopsy in this subgroup, yet inconsistencies remain, warranting further research [6, 9, 10]. This becomes even more pertinent in low-resource settings, where access to advanced diagnostic tools is limited.

Thus, the present study was designed to explore the histopathological spectrum of endometrial findings in peri- and postmenopausal women presenting with AUB, while simultaneously evaluating the utility of TVS-derived ET as a predictive marker for premalignant and malignant lesions. By correlating imaging findings with histology, this study aims to enhance diagnostic confidence, reduce unnecessary interventions, and contribute to more tailored patient care.

## Methods

This was a retrospective, observational cohort study conducted at the Department of Pathology, Kasturba Medical College, Manipal. The study spanned a 12-month period from January 2021 to December 2021. Ethical clearance was obtained from the Institutional Ethics Committee prior to initiation of the study. All relevant clinical data, endometrial thickness measured by transvaginal ultrasonography and histopathological slides were retrieved from departmental archives and hospital electronic databases.

A total of 455 endometrial samples were received in the department during the study period. Following stringent inclusion and exclusion criteria, 371 cases were shortlisted for final analysis. Women included in the study were either perimenopausal (aged approximately 40–50 years with irregular cycles) or postmenopausal (defined as cessation of menstruation for ≥ 12 months) and had presented with abnormal uterine bleeding.

### Inclusion criteria

- Women aged ≥ 40 years.
- Presenting with AUB.
- Availability of both transvaginal sonographic data and histopathological slides.

### Exclusion criteria

- Inadequate endometrial sample.
- Hysterectomy specimen.
- Endometrial sample received from other outside hospitals where clinico-radiological data was not available.

Patient demographics including age, menopausal status, parity, and clinical presentation were recorded. Special attention was paid to drug history, particularly hormone replacement therapy (HRT) or tamoxifen use. Transvaginal ultrasonography (TVS) findings were reviewed with specific focus on endometrial thickness (ET), which was recorded in millimeters as the maximum anteroposterior thickness in a longitudinal section of the uterus. All histopathological slides for each case were studied individually by two pathologists and morphological parameters were assessed.

### Statistical analysis

Quantitative data were entered in Microsoft Excel and analyzed using SPSS software version 27.0. Categorical and continuous variables were reported in proportions and mean with standard deviation. Endometrial thickness was treated as a continuous variable, and mean ET values were compared across histological subgroups using Mann-Whitney U Test. Sensitivity, specificity, area under curve (AUC), and diagnostic cut-off values for ET in predicting hyperplasia and carcinoma were calculated using receiver operating characteristic (ROC) analysis. Chi square or K-Fischer test (if expected count < 5) was used to assess the association between the variables and study groups. The statistical significance was determined at a 5% level of significance.

## Results

The study analysed a total of 307 cases of abnormal uterine bleeding (AUB) in women aged 40 years and above. Among these, 79.2% were categorized as perimenopausal, 19.5% as postmenopausal based on menstrual history. Menopause status was not known in 1.3% cases. Menorrhagia was the most common symptom, followed by postmenopausal bleeding and dysmenorrhea.

**Fig. 1:**
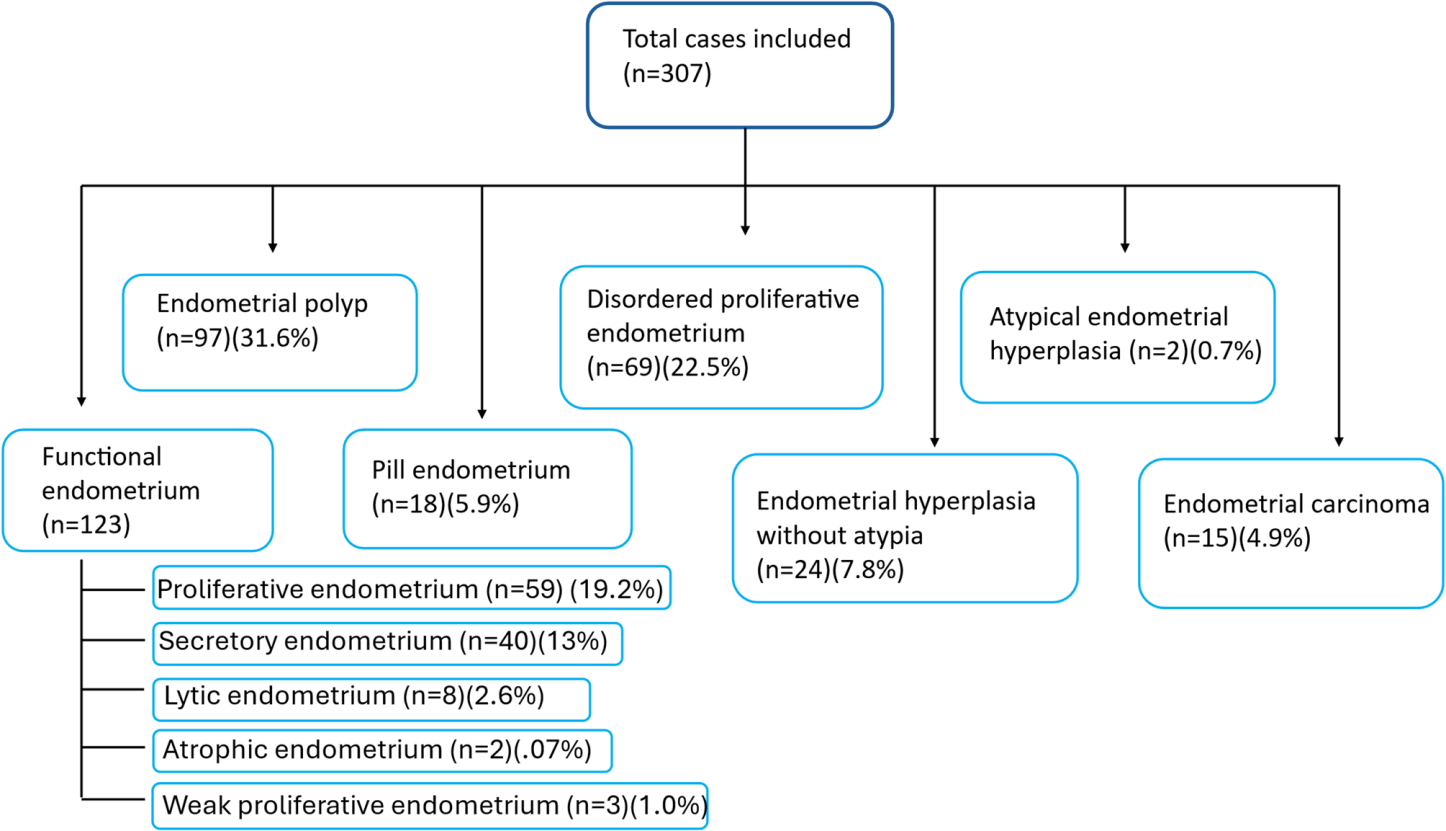
Flow diagram of histomorphology

Endometrial sampling was obtained by curettage and biopsy. 206/307 samples were obtained by curettage and remaining 47/307 samples through biopsy. It revealed a diverse histomorphological spectrum as shown in Fig. 1. In the perimenopausal group, the most frequently encountered patterns included endometrial polyp (*n* = 70)), disordered proliferative endometrium (*n* = 64) and proliferative endometrium (*n* = 53). In contrast, the postmenopausal group showed a predominance of endometrial polyp (*n* = 25), atrophic endometrium (*n* = 11) and endometrial carcinoma (*n* = 11) (Table 1). Various forms of metaplasia were observed, including tubal metaplasia, eosinophilic metaplasia, and squamous morular metaplasia, particularly in patients with hyperplasia or hormone exposure.

**Table 1:**
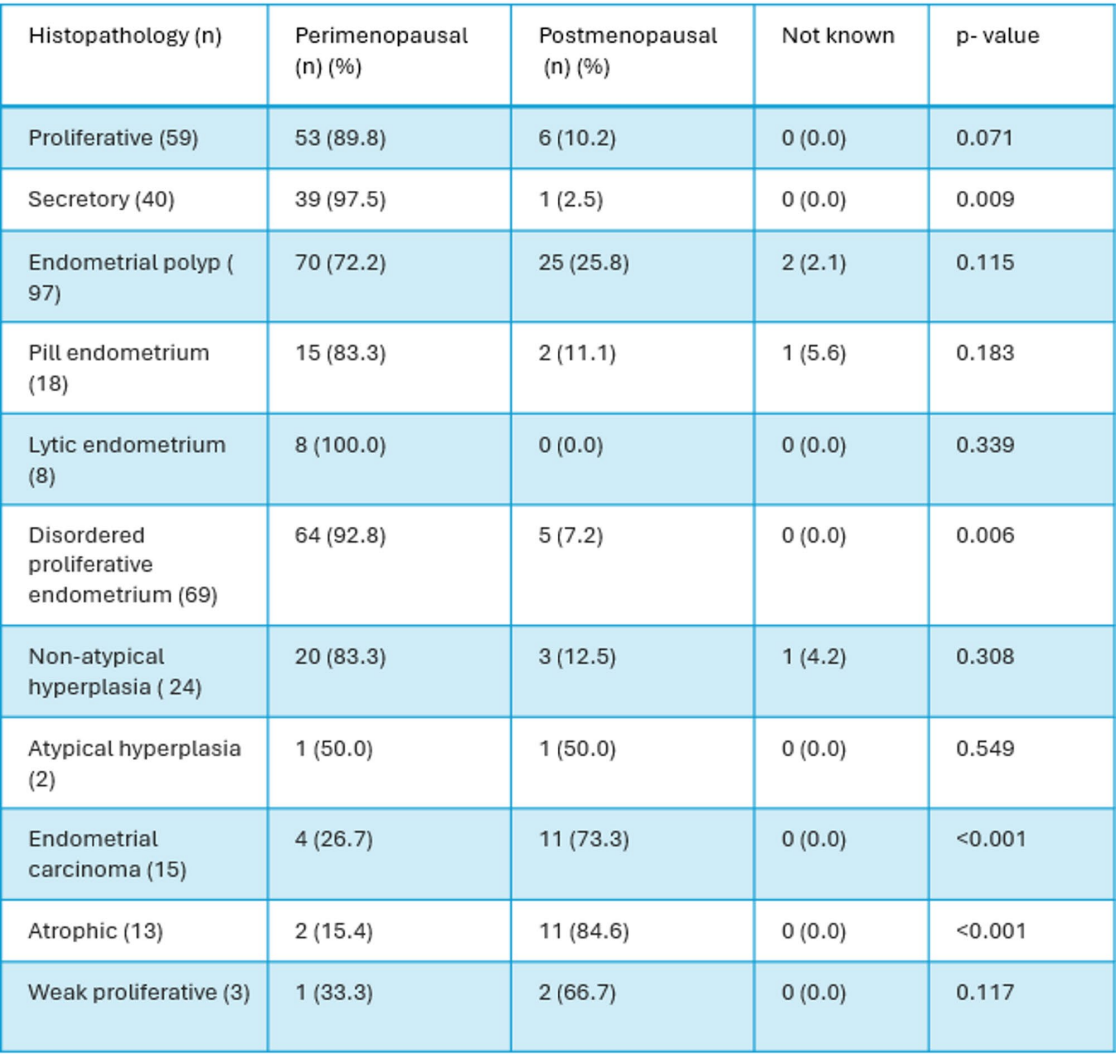
Histomorphological spectrum of endometrium in Peri and post-menopausal women.

In this study, we have categorized endometrial thickness (ET) into six groups as shown in Table 2:

- < 5 mm.
- 5–10 mm.
- 11–15 mm.
- 16–20 mm.
- 21–25 mm.
- 26–30 mm.
- > 30 mm.

Most of the functional endometrium had ET ranging between 5 and 10 mm. Among the functional causes, proliferative endometrium was the most commonly seen histomorphology constituting 50.8%. Endometrial Polyps frequently occur with ET ranging between 5 and 10 mm (44.3%) and 11–15 mm (29.9%). It was found that a significant proportion of non-atypical hyperplasia had an ET ranging between 16 and 20 mm and is significantly associated with an ET of 16–20 mm constituting 45.8%. A notable case of endometrial carcinoma had ET ranging between 11 and 15 mm. Endometrial carcinoma cases increase with ET, with notable cases at 11–15 mm and 21–25 mm and correlates with increasing ET. 2 cases constituting 1.6% of endometrial carcinoma showed ET in the range of 5–10 mm. Higher ET is significantly associated with hyperplasia and carcinoma and shows strong statistical significance (*p* < 0.001). Lower ET (< 5 mm) is more common in benign conditions like endometrial polyp and proliferative endometrium.

**Table 2:**
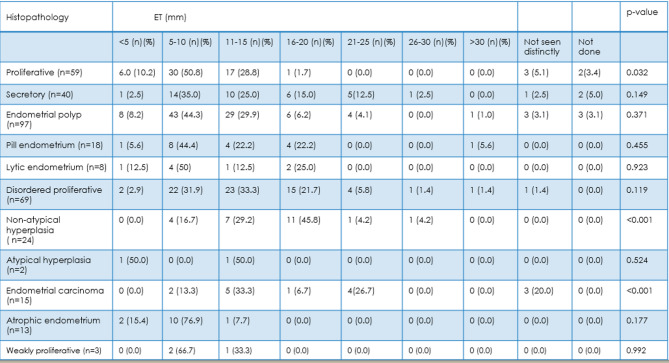
Correlation of endometrial thickness (mm) among various histomorphological spectrum.

Use of tamoxifen and HRT may influence the ET measurements. Hence, in this study, the role of such confounding factors was separately analysed. However, the status of HRT and tamoxifen were not known for 219/307 cases. 5 patients had a known history of tamoxifen use, out of which 2 were diagnosed with endometrial polyp, 1 proliferative endometrium, 1 weak proliferative endometrium and 1 atrophic endometrium. None of the patients with tamoxifen history had pre-malignant or malignant pathology in the endometrium on H&E evaluation. Hence, none had interfered with determining the ET cut-off in detecting pre-malignant and malignant pathology. 44 patients had known history of use of HRT and most of them were diagnosed with endometrial polyp and pill endometrium on H&E evaluation.

**Fig. 2:**
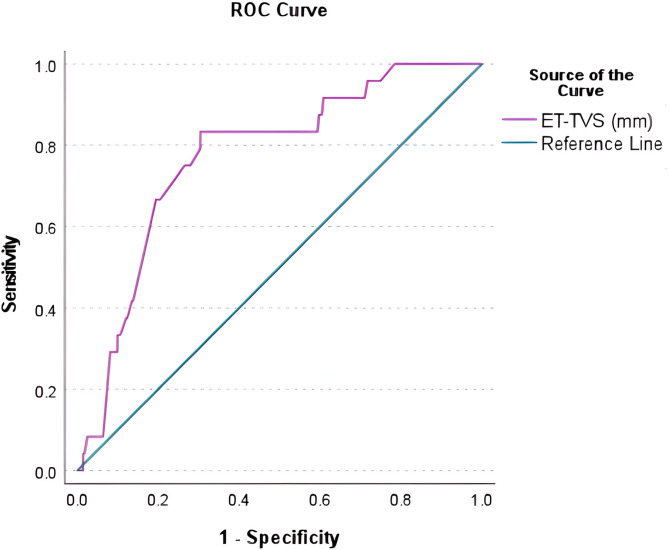
Receiver operating characteristic (ROC) curve for TVUS-ET values obtained from all cases of non-atypical hyperplasia in peri and post-menopausal women with AUB.

Receiver operating characteristic (ROC) curve analysis (Fig. 2) was performed to determine the diagnostic utility of ET in detecting premalignant and malignant endometrium in perimenopausal and post-menopausal patients with AUB. In our study, (*n* = 307) the cut-off of ET in non-atypical hyperplasia (*n* = 22); the area under the curve (AUC) (Table 3) was 0.772 with a sensitivity of 83.3%, specificity of 69.6% and the cut-off was found to be > 12.85 mm. In atypical hyperplasia (*n* = 2), AUC couldn’t be calculated as the sample size was low (only 2 cases). In endometrial carcinoma, the AUC = 0.691 with a sensitivity of 93.3%, specificity 42.1% & cut-off ≥ 9.85 mm.

**Table 3:** Table showing sensitivity, specificity, AUC & cut-off of ET in non-atypical endometrial hyperplasia in Peri and post-menopausal women with AUB.

AUC	Sensitivity	Specificity	Cut-off
0.772	83.3	69.6	≥ 12.85

To study the endometrial thickness among various histological types of endometrial carcinoma, we stratified the carcinoma into subtypes (type I and type II) and then correlated with the endometrial thickness (Table 4). Out of the 15/307 cases of endometrial carcinoma,

TYPE I: Endometrioid endometrial carcinoma: -.

- FIGO grade I: 7 cases.
- FIGO grade II: 1 case.
- FIGO grade III: 5 cases.

TYPE II: 

 -Clear cell carcinoma: 1 case.

-High grade serous carcinoma: 1 case.

**Table 4:** Carcinoma subtypes and its correlation with ET (mm).

TYPE I	HISTOLOGY TYPE	ET (mm)				
		**< 5**	**5–10**	**11–15**	**16–20**	**21–25**
	• EEC: FIGO grade I	0	1	3	0	3
	• EEC: FIGO grade II	0	0	1	0	0
	• EEC: FIGO grade III	0	1	1	1	1
TYPE II	• Clear cell carcinoma	0	0	1	0	0
	• High grade serous carcinoma	0	0	0	0	1

It was found that the two cases which had ET between 5 and 10 mm, did not exhibit any aggressive histology. Both the cases were EEC, FIGO grade I and grade III respectively.

These findings underscore the clinical value of ET measurement via TVS as a non-invasive screening modality that correlates well with underlying histological changes. The data also reinforce the need for biopsy in patients with high ET values, especially in those who are postmenopausal.

## Discussion

Abnormal uterine bleeding (AUB) continues to be a significant diagnostic and therapeutic challenge in gynecologic practice, particularly among women transitioning through perimenopause and those in the postmenopausal phase. This study explored the histomorphological spectrum of endometrial changes in such women and established a meaningful correlation with endometrial thickness (ET) measured through transvaginal sonography (TVS).

The findings reaffirm that AUB, while often benign in origin, can occasionally be a harbinger of serious underlying pathology, including endometrial hyperplasia and carcinoma. The perimenopausal group in our study primarily presented with menorrhagia and was histologically dominated by endometrial polyp, disordered proliferative endometrium and proliferative endometrium—findings consistent with studies by Damle et al. and Sharma et al., who reported similar endometrial profiles among women in this transitional phase of hormonal flux [11, 12]. On the other hand, postmenopausal women in our cohort most frequently exhibited endometrial polyp and atrophic endometrium, aligning with the results of Veena et al. and Hussain et al., which underscore atrophic changes as the predominant histological feature in the absence of hormonal cycling [13, 14].

Endometrial hyperplasia without atypia was found more in perimenopausal women (83.3%). This supports the role of unopposed estrogen stimulation, which is more common in anovulatory cycles seen during the menopausal transition. These findings correlate with the observations made by Thulasi et al. and Saravade et al., where simple hyperplasia represented a substantial fraction of endometrial pathology in women with AUB [10, 15]. In perimenopausal women, although hormonal cycling creates variability in ET, our findings suggest that a threshold of 11–12 mm still holds significant predictive value for hyperplasia and carcinoma, consistent with studies by Kumari et al. and Vani et al. [6, 16].

Interestingly, cases of endometrial carcinoma were predominantly seen in postmenopausal women and were associated with ET values >11 mm. This supports the widely held view that endometrial cancer in postmenopausal women is usually preceded by a marked increase in endometrial thickness and justifies the approach of prioritizing endometrial biopsy in such cases regardless of symptom severity. These observations are consistent with the diagnostic pathway advocated by the FIGO classification system, which recommends invasive sampling in high-risk settings [5]. However, according to our study, ROC cut-off for endometrial carcinoma was found to be ≥ 9.85 mm, however 2 cases constituting 1.6% of endometrial carcinoma showed ET in the range of 5–10 mm and none of the cases had ET < 5 mm. Therefore, in the postmenopausal subset, an ET threshold of 4.5 mm reliably differentiated benign from potentially malignant lesions highlighting that in postmenopausal women with ET >5 mm and bleeding, biopsy remains imperative even if TVS appears normal. All carcinoma cases in this group exceeded this cut-off, emphasizing the utility of the 4–5 mm benchmark suggested by ACOG guidelines as an appropriate threshold for further investigation [8].

Metaplastic changes, although not diagnostic on their own, were frequently observed in conjunction with hormone-induced alterations and hyperplasia. The high prevalence of tubal metaplasia and eosinophilic changes describe these alterations as secondary responses to estrogenic stimulation or tissue injury [17, 18].

While transvaginal ultrasonography is widely accepted as a non-invasive screening tool, it is not without limitations. Factors such as obesity, uterine fibroids, and prior surgeries can hinder accurate assessment of endometrial echo. Nevertheless, the strong statistical correlation between ET and histological abnormalities in this study supports its continued use as a first-line modality.

This study’s strength lies in its cohort-based approach with histopathological validation, which enhances the reliability of findings. However, limitations include its retrospective design, single-centre setting, and exclusion of molecular profiling, which could provide deeper insight into the pathogenesis of endometrial changes.

In summary, this study emphasizes that while AUB is often functional in origin, its evaluation requires a systematic and cautious approach. One should also keep in mind about the potential inter-observer variability in diagnosing atypical hyperplasia among pathologists. The combination of clinical evaluation, TVS-derived ET measurement, and histopathological confirmation remains the gold standard for diagnosis, particularly in postmenopausal women where the risk of malignancy is higher. Early detection through appropriate thresholds can significantly reduce morbidity and enable timely intervention, particularly in resource-constrained settings.

## Limitations

1. Retrospective design – the study is subject to limitations inherent in retrospective analysis such as missing data and selection bias.
2. Sampling variability – the diagnostic yield of endometrial curettage and biopsy may miss focal lesions due to sampling errors.
3. Use of tamoxifen and HRT may influence the ET measurements. However, due to the retrospective nature of the study, it was impossible to adjust these confounding factors or to incorporate multivariate analysis, since data was not available in majority of the patients.
4. The study’s retrospective design made it impossible to determine the exact menstrual phase during which the ET was measured in perimenopausal women. Since endometrial thickness varies during the course of a woman’s menstrual cycle, in women with atypical and cyclical bleeding, a standardization in timing of ultrasound procedure will prevent misleading interpretation of thick endometrium.
5. Small number of malignant and atypical cases – limited sample size in these categories restricts the ability to derive strong statistical conclusions.

## Conclusion

This study reinforces the pivotal role of histopathological evaluation in the diagnostic workup of abnormal uterine bleeding (AUB) in peri- and postmenopausal women and highlights that it is mostly associated with benign conditions like polyps and disordered proliferative endometrium. However, over 13% of patients with AUB had premalignant or malignant endometrial pathology, underscoring the importance of endometrial evaluation, especially when endometrial thickness exceeds diagnostic cutoffs.

Transvaginal ultrasonography (TVS) proved to be an effective, non-invasive preliminary tool. The study found that an ET threshold of 11–12 mm in perimenopausal women and > 5 mm in postmenopausal women could reliably predict the likelihood of premalignant or malignant pathology. These values support current clinical practice guidelines and also suggest avenues for refining decision-making protocols, especially in resource-limited settings. In postmenopausal women with ET > 5 mm and bleeding, biopsy remains imperative even if TVS appears normal.

This study underscores the importance of individualized, evidence-based assessment of AUB and highlights how simple diagnostic tools, when applied judiciously, can significantly improve patient outcomes through early detection and intervention.

## Data Availability

No datasets were generated or analysed during the current study.

## Abbreviations

ACOG: American College of Obstetricians and Gynecologists
AUB: Abnormal Uterine Bleeding
AUC: Area Under Curve
ET: Endometrial thickness
FIGO: International Federation of Gynecology and Obstetrics
HRT: Hormone Replacement Therapy
ROC: Receiver Operating Characteristic Curve
TVS: Trans Vaginal Sonography

## References

[1] Karimi M, Alizadeh A, Mahmoodi M. Clinicopathological pattern of endometrial specimens in women with abnormal uterine bleeding and ultrasonography correlation. Arch Iran Med. 2024;27(4):216–22. 38685848 10.34172/aim.2024.31 PMC11097308

[2] Chhatrasal C, Shelgaonkar G, Ghanghoria VK, Yadav S, Aggarwal A. Evaluation of endometrial histopathological patterns in abnormal uterine bleeding: a study of 1545 cases. Int J Med Sci Public Health. 2017;6(7):1.

[3] Papakonstantinou E, Adonakis G. Management of pre-, peri-, and post menopausal abnormal uterine bleeding: when to perform endometrial sampling? Int J Gynecol Obstet. 2022;158:252–9. 10.1002/ijgo.13988 34669187

[4] Pennant ME, Mehta R, Moody P, Hackett G, Prentice A, Sharp SJ, et al. Premenopausal abnormal uterine bleeding and risk of endometrial cancer. BJOG. 2017;124(3):404–11. 27766759 10.1111/1471-0528.14385 PMC5297977

[5] National Heart, Lung, and Blood Institute. Management of acute abnormal uterine bleeding in nonpregnant reproductive-aged women *Internet+. Available from: http://www.nhlbi.nih.gov/guidelines/vwd/vwd.pdf

[6] Kumari P, Gaikwad HS, Nath B. Endometrial cut-off thickness as predictor of endometrial pathology in perimenopausal women with abnormal uterine bleeding: A cross-sectional study. Obstet Gynecol Int. 2022;2022. 10.1155/2022/5073944 PMC8752292 35027929

[7] Nazim F, Hayat Z, Hannan A, Ikram U, Nazim K. Role of transvaginal ultrasound in identifying endometrial hyperplasia. J Ayub Med Coll Abbottabad. 2013;25(1 2):100–2. 25098067

[8] The role of. transvaginal ultrasonography in evaluating the endometrium of women with postmenopausal bleeding.

[9] Giannella L, Cerami LB, Setti T, Bergamini E, Boselli F. Prediction of endometrial hyperplasia and cancer among premenopausal women with abnormal uterine bleeding. Biomed Res Int. 2019;2019. 10.1155/2019/8598152 PMC6442314 31011581

[10] Correlation of endometrial thickness by. trans-vaginal sonography and histopathology in women with abnormal peri-menopausal and postmenopausal bleeding – A prospective study. Indian J Obstet Gynecol Res. 2020;5(1):44–8.

[11] Damle RP, Dravid NV, Suryawanshi KH, Gadre AS, Bagale PS, Ahire N. Clinicopathological spectrum of endometrial changes in peri-menopausal and post-menopausal abnormal uterine bleeding: a 2 years study. J Clin Diagn Res. 2013;7(12):2774–6. 24551634 10.7860/JCDR/2013/6291.3755 PMC3919318

[12] Sharma R, Mishra P, Kumar N, Srivastava P. Histomorphological spectrum of endometrial lesion in women presenting with abnormal uterine bleeding: A 3 year study at a tertiary care center. Trop J Pathol Microbiol. 2018;525. Available from: www.pathologyreview.in.

[13] Veena P, Baskaran D, Maurya DK, Kubera NS, Dorairaj J. Addition of power doppler to grey scale transvaginal ultrasonography for improving the prediction of endometrial pathology in perimenopausal women with abnormal uterine bleeding. Indian J Med Res. 2018;148(3):302–8. 30425220 10.4103/ijmr.IJMR_96_17 PMC6251271

[14] Husain S, Al Hammad RS, Alduhaysh AK, AlBatly MM, Alrikabi A. Pathological spectrum of endometrial biopsies in Saudi women with abnormal uterine bleeding: A retrospective study of 13 years. Saudi Med J. 2021;42(3):2709. 10.15537/smj.2021.42.3.20200814 PMC7989265 33632905

[15] Saravade VR, Chaturvedi S. Study of endometrium by trans-vaginal sonography and its correlation with histopathology in perimenopausal women with abnormal uterine bleeding. Int J Reprod Contracept Obstet Gynecol. 2021;10(9):3554.

[16] Bai SVB. JP. Histopathological evaluation of endometrial biopsies and curettings in abnormal uterine bleeding. 2019. Available from: www.medresearch.in.

[17] Goldblum JR, McKenney KJ, Lamps LW, Myers JL. Rosai & Ackerman’s surgical pathology. 3rd ed. 2020;2:1260–94.

[18] Nicolae A, Preda O, Nogales FF. Endometrial metaplasias and reactive changes: A spectrum of altered differentiation. J Clin Pathol. 2011;64:97–106. 21126963 10.1136/jcp.2010.085555

